# Successful Implementation of Newborn Screening for Hemoglobin Disorders in the Philippines

**DOI:** 10.3390/ijns7020030

**Published:** 2021-06-17

**Authors:** Carmencita D. Padilla, Bradford L. Therrell, Maria Melanie Liberty B. Alcausin, Reynaldo C. de Castro, Maria Beatriz P. Gepte, Ma. Elouisa L. Reyes, Charity M. Jomento, Riza Concordia N. Suarez, Ebner Bon G. Maceda, Conchita G. Abarquez, J. Edgar Winston C. Posecion, Alma P. Andal, Anna Lea G. Elizaga, Bernadette C. Halili-Mendoza, Maria Paz Virginia K. Otayza, Carolyn C. Hoppe

**Affiliations:** 1 Newborn Screening Reference Center, National Institutes of Health, University of the Philippines Manila, Manila 1000, Philippines; mbalcausin@up.edu.ph (M.M.L.B.A.); rdcjpcmc@yahoo.com (R.C.d.C.J.); docbea20@yahoo.com (M.B.P.G.); mlreyes9@up.edu.ph (M.E.L.R.); cmjomento@up.edu.ph (C.M.J.); rnsuarez@up.edu.ph (R.C.N.S.); egmaceda@up.edu.ph (E.B.G.M.); 2 Department of Pediatrics, College of Medicine, University of the Philippines Manila, Manila 1000, Philippines; 3 National Newborn Screening and Global Resource Center, Austin, TX 78759, USA; therrell@uthscsa.edu; 4 University of Texas Health Science Center at San Antonio, San Antonio, TX 78229, USA; 5 Newborn Screening Center—Mindanao, Southern Philippine Medical Center, Davao 8000, Philippines; conch.abarquez@gmail.com; 6 Newborn Screening Center—Visayas, West Visayas State University Medical Center, Iloilo City 5000, Philippines; eposecion@gmail.com; 7 Newborn Screening Center—Southern Luzon, Daniel O. Mercado Medical Center, Tanauan City 4232, Philippines; ampanganiban@dmmcinc.com; 8 Newborn Screening Center—National Institutes of Health, Quezon City 1101, Philippines; agelizaga@up.edu.ph; 9 Newborn Screening Center—Central Luzon, Angeles City University Foundation Medical Center, Angeles City 2009, Philippines; bernadettehmendozansc@gmail.com; 10 Newborn Center—Northern Luzon, Mariano Marcos Memorial Hospital and Medical Center, Batac City 2906, Philippines; bingotayza@gmail.com; 11 UCSF Benioff Children’s Hospital Oakland, Oakland, CA 94609, USA; choppe@gbt.com; 12 Global Blood Therapeutics, Inc., South San Francisco, CA 94080, USA

**Keywords:** newborn screening, thalassemia, hemoglobinopathy, HPLC, Philippines

## Abstract

The Philippine newborn bloodspot screening (NBS) program began in 1996 with 24 hospitals and was formalized by legislation in 2004. The NBS panel was recently expanded to include a number of additional hereditary congenital conditions. Expertise and experiences from other NBS programs already screening for hemoglobinopathies were essential to its successful integration into the ongoing dried bloodspot NBS program in the Philippines. Building on clinical experiences and population data from Filipinos born in California, USA, hemoglobinopathies (including thalassemias) were selected for inclusion in the expanded screening panel. Hemoglobinopathy NBS, using high performance liquid chromatography, was implemented in a stepwise manner into the seven regional NBS screening laboratories. A central university laboratory provides confirmatory testing using both capillary electrophoresis and molecular methodologies. NBS results indicating carriers are followed up with educational fact sheets, while results of presumptive disease are referred for confirmatory testing and follow-up with a hematologist. Long-term care is provided through newborn screening continuity clinics across the country. Hemoglobinopathy NBS is now included in the national insurance package and screening uptake continues to increase nationally, exceeding 90% of all newborns in 7400+ hospitals and birthing centers nationwide prior to the COVID-19 pandemic.

## 1. Introduction

### 1.1. Evolution of Newborn Screening in the Philippines

Newborn bloodspot screening (NBS) began in the Philippines in 1996 as a research pilot in 24 hospitals, accredited by both the Philippine Pediatric Society (PPS) and the Philippine Obstetrical and Gynecological Society (POGS), in Metro Manila. The initial intent was: (1) to establish the incidence of 6 congenital conditions, congenital hypothyroidism (CH), phenylketonuria (PKU), galactosemia (GAL), congenital adrenal hyperplasia (CAH), homocystinuria (HCY), and glucose-6-phosphate dehydrogenase (G6PD) deficiency; and (2) to make recommendations for the adoption of newborn screening nationwide. Since funding was not available, a small fee (approximately USD 9) was required [1]. Building on the data obtained, a 5-condition screening panel, excluding G6PD deficiency, was initiated in 1996 funded by the USD 9 fee-for-service [1,2,3]. NBS for G6PD deficiency was added to the screening panel in 1998. NBS services for other hospitals (generically referred to as Newborn Screening Facilities or NSFs) was opened in 1999 and, by 2000, 153 hospitals were providing NBS, 114 of which were outside of Metro Manila [1]. In 2000, HCY was discontinued due to a lack of case findings and later, in 2012, maple syrup urine disease (MSUD) was added to the screening panel, completing the basic panel in place prior to the most recent expansion discussed here [4].

Coordination of NBS laboratory services with the New South Wales, Australia NBS program was essential during early program development. For 15 months in 1996–1997, specimens were sent daily from Manila, Philippines to Sydney, Australia while the first Philippine NBS laboratory was being developed [1]. Technical guidance from the University of the Philippines Manila, various supporting administrative orders from the Department of Health (DOH), inclusion of NBS in the DOH Child Health 2025 plan, and a Philippine Presidential proclamation in 2003 were some of the key elements contributing to successful adoption of the National Screening Act of 2004 (Republic Act No. 9288), which required that NBS be offered to all newborns [3,5,6]. In 2006, the fee for the 5-condition NBS panel in place at the time was included as a newborn health benefit by national health insurance (PhilHealth). Combined, the law and national insurance significantly improved national NBS coverage [7].

As the Philippine NBS system matured (2000–2006), increased knowledge in medical genetics and significant improvements in screening technology in developed NBS programs, particularly in the U.S., led to expanded screening panels that allowed for early detection and treatment of clinically significant diseases in an increasing number of newborns [8,9]. The consideration of possible NBS expansion, and related projects to accumulate incidence data on screenable conditions in the Philippines, led to a search for data from existing expanded NBS programs in regions with large populations of Filipino expatriates. In particular, data from the California, USA NBS program (CNSP) showed a high prevalence of, and ability to detect, clinically significant hemoglobin disorders (including thalassemias) in Filipino newborns [10,11].

### 1.2. Hemoglobin Disorders

Hemoglobinopathies are a group of inherited disorders characterized by either structural variants in the alpha (α)-globin and beta (β)-globin genes or quantitative differences in α- or β-globin chain production, which can have severe clinical significance [12]. Sickle cell disease (SCD), for example, refers to a group of hemoglobinopathies caused by structural variations in the β-globin chain. On the other hand, decreased amounts of α- or β-globin chains results in α- or β-thalassemia, respectively. While traditional hemoglobinopathy NBS programs, particularly in the U.S. and Europe, have focused on detecting SCD, clinically significant thalassemias are also detectable either directly from NBS or indirectly as a consequence of the differential diagnosis resulting from NBS [13,14,15]. Various NBS laboratory testing techniques for hemoglobinopathies are commercially available and programs usually select their methodology or methodologies based on cost, ease of use, and detection efficiency [12,16].

Thalassemias, the most common human monogenic diseases, have a high prevalence in Asian populations. While β-thalassemias, in general, are clinically more significant, α-thalassemias, with their variable phenotypes, have a higher overall frequency. The most severe clinical phenotype of β-thalassemia, β-thalassemia major, can result from any one of several causes, including the complete absence of β-globin chains (β^0^-thalassemia) and co-inheritance of a β-thalassemia mutation and hemoglobin (Hb) E, a common β-globin variant in Asians, (Hb E/β-thalassemia). Unable to produce normal hemoglobin, children with β-thalassemia major develop severe anemia, which requires lifelong transfusions, and results in massive hepatosplenomegaly, poor growth, and skeletal abnormalities.

The clinical severity of α-thalassemias is directly related to the type and number of α-globin gene mutations involved. Individuals with one or two α-globin gene deletions or mutations are asymptomatic, while deletion or inactivation of three α-globin genes results in Hb H disease, and deletion of all four α-globin genes results in Hb Bart’s hydrops fetalis, which is usually fatal in utero or at birth. Hb H disease is usually characterized by hemolytic anemia and is more severe in individuals with coinheritance of two α-globin gene deletions and Hb Constant Spring, another fairly common mutation in the Southeast Asian population (Hb H/Constant Spring). Individuals affected with Hb H disease are usually asymptomatic at birth [17].

Like SCD, thalassemias can be detected through NBS and related confirmatory studies, and early diagnosis and treatment has been shown to significantly improve health outcomes [13,17,18]. The prevalence of thalassemias in the Philippine population is not accurately known. Limited population studies of Filipinos in Hawaii and Taiwan found α-gene carrier frequencies of 9.1% and 6.8%, respectively [19,20]. Another Taiwan study showed a β-gene carrier frequency of 0.9% in Filipinos [21].

This report reviews the selection of hemoglobinopathies as part of the Philippine NBS panel of disorders, and the successful integration of hemoglobinopathy NBS into an ongoing screening program in a developing economy, with the hope that it will provide valuable information for other developing or expanding NBS programs in similar circumstances.

## 2. Methodology

Significant advances in screening techniques and disease knowledge towards the end of the 20th century led to reconsiderations of the disorders included in NBS panels in both the U.S. and Europe, among others [8,9,22,23]. This included the methods for selecting conditions for inclusion on the screening panel and various other expansion considerations (public health impact, cost, and screening efficacy, etc.) Gaining from these experiences, the Philippine NBS program initiated a review of its options regarding expansion.

For any new disorder, pilot studies to determine its prevalence are significant challenges to initiating expanded screening as they can be both time consuming and expensive. Alternatively, a review of available data from NBS programs screening large numbers of Filipino expatriates was initiated in order to ascertain: (1) whether NBS results on disorders not currently included in the Philippine NBS program were available, and (2) whether any such results were suitable to serve as a proxy for pilot testing in the Philippines. Preliminary data obtained from the CNSP (published later) initially provided information on over 60,000 Filipino newborns screened in California [10]. Additional data increased the pool of screened Filipino births to over 110,000 and these combined data were ultimately used to evaluate the addition of hemoglobinopathies to the Philippine NBS panel [11].

Screening laboratory techniques were a major consideration (due to lack of local knowledge and commercial product support) and were focused on the expectation that α-thalassemias would be the primary disorders identified based on both CNSP data and local clinical experiences [11]. Intensive discussions with international experts experienced in hemoglobinopathy NBS provided broad input into planning and strategies for overcoming the potential challenges of program expansion. Experiences from other hemoglobinopathy NBS programs, predominantly in the U.S. where hemoglobinopathy NBS has the longest history [24], were reviewed to evaluate the strengths and weaknesses of available screening laboratory methodologies, proper flow within the laboratory, and reporting, tracking, and follow-up protocols. Published experiences also provided information on whether and how to use a central confirmatory laboratory, and the strengths and weaknesses of various confirmatory laboratory methodologies [25,26].

To aid in defining laboratory and follow-up processes, a hemoglobinopathy NBS algorithm was developed. As the CNSP includes several screening laboratories and follow-up centers, similar in many respects to the Philippine NBS system defined elsewhere [27], the CNSP screening flow was used as a template and modified to accommodate local circumstances. A Philippine-based pediatric hematologist was identified to serve as the professional lead and consultant for the program. In order to better understand the issues that might be encountered upon start-up in the Philippines, an orientation visit to the CNSP was arranged.

**Figure 1:**
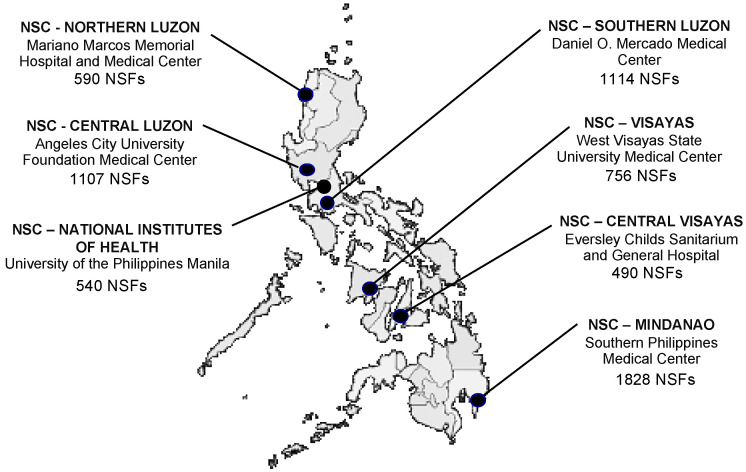
Newborn Screening Centers (NSCs) currently providing screening laboratory services throughout the Philippines and the number of Newborn Screening Facilities (NSFs) collecting and submitting screening specimens.

To begin developing a hemoglobinopathy knowledge base in the Philippines, educational workshops were organized for Philippine stakeholders. Initially, international experts were invited to conduct a workshop on implementing an NBS program for hemoglobinopathies. This first workshop was attended by local pediatric hematologists, NBS center (NSC) heads and laboratory managers (at the time, there were six NSCs in the country, see Figure 1), and other program support staff (including selected administrative, laboratory, follow-up, and quality assurance personnel). Faculty for this two-day workshop included two international experts in NBS and pediatric hematology, the local hematologist and program consultant, and local NBS program administrators. The workshop content included basic medical information on hemoglobinopathies and thalassemias, international NBS experiences with hemoglobinopathy testing and follow-up, and potential challenges in implementing hemoglobinopathy NBS in the Philippines.

Subsequently, additional workshops were conducted by local pediatric hematologists and other members of the NBS teams in the various NSCs. Conference calls with the international experts were conducted as issues requiring their input or clarification arose. In addition to the orientation visit of the hematologist to the CNSP, study visits were also made by the Philippine NBS program director and the quality assurance officer. Immediately prior to screening implementation, a refresher workshop and review of the screening algorithms was conducted by the international experts with attendees limited to NBS program personnel involved directly in hemoglobinopathy expansion. Workshop faculty also included an expert from the laboratory instrument manufacturer with expertise in both NBS and hematology.

An education and information sharing plan was developed to inform physicians, parents, and other stakeholders, including the national insurance provider, about the impending program expansion. A phased-in laboratory approach was also planned, which included consideration of other conditions being simultaneously added to the screening panel and targeted for implementation at about the same time.

## 3. Results

A review of published and unpublished data from the CNSP revealed that in a population of 111,127 Filipino newborns, 199 were identified with disorders included on the CNSP panel. Of these, 109 were confirmed to have a hemoglobin disorder. The predominant finding was Hb H disease, with a prevalence of 76.1 per 100,000 births in Filipinos [10]. The combined hemoglobinopathy data from the CNSP were used to estimate prevalence in the newborn population of the Philippines, assuming an annual birth cohort of 2 million newborns (see Table 1) [11]. Of particular concern was the relatively high prevalence of Hb H disease, which was estimated to exceed the prevalence of any other screened disease in the Philippines with the exception of G6PD deficiency. Emphasis on expanded hemoglobinopathy screening, therefore, focused on understanding and detecting α-thalassemias.

The evaluation of the various screening laboratory methodologies revealed a slightly better resolution and quantification of Hb Bart’s, the abnormal form of hemoglobin in newborns indicative of possible α-thalassemia, using high performance liquid chromatography (HPLC); this methodology was thus chosen as the primary screening technique for implementation in all NBS centers [28]. While capillary electrophoresis (CE) was given strong consideration as a primary screening laboratory methodology [29], a commercially available NBS product using dried bloodspot samples of the same diameter used for testing in the Philippine NBS program was not available at the time. The confirmatory laboratory, however, uses CE initial testing for NBS confirmation prior to any molecular testing (see discussion below).

A single NSC was chosen to order and install equipment, add necessary personnel, procure supplies, undergo personnel training, and begin screening (a process taking approximately 18 months). The U.S. Centers for Disease Control and Prevention (CDC) Newborn Screening Quality Assurance Program (NSQAP) [30] was contacted and they agreed to provide limited numbers of quality control (QC) and proficiency testing (PT) specimens to assist in laboratory implementation. The validation of the laboratory testing protocol included satisfactory analysis of approximately 2000 specimens prior to implementation.

Once validated, increasing numbers of specimens were tested until all specimens received daily were being tested (approximately 2 weeks). The program’s quality assurance officer confirmed that the courier system already in place between NSFs and NSCs provided timely specimen transport and included environmental safeguards sufficient to preclude specimen degradation due to specimen age or heat. Successful integration of hemoglobinopathy testing into the first screening laboratory (NSC-National Institutes of Health, Quezon City) in December 2014 was followed by successful integration into NBS activities in: NSC-Visayas, Iloilo City in November 2015; NSC-Central Luzon, Angeles City in January 2016; NSC-Mindanao, Davao in July 2017; NSC-Southern Luzon, Tanauan City in July 2018; and Northern Luzon, Batac City in January 2019 (Figure 1). In the interim, while hemoglobinopathy screening was being implemented, another NSC opened in Cebu, Philippines (NSC-Central Visayas) and hemoglobinopathy screening was initiated there in February 2020. In each case, approximately 2000 specimens (including patients, NSQAP QC and PT specimens, and manufacturer’s controls) were required to be satisfactorily analyzed in parallel with another NSC before routine testing was initiated. All NSCs were enrolled in the NSQAP quarterly PT program and their performance, evaluated as part of DOH laboratory certification, included their compliance with the Philippine Performance Evaluation and Assessment Scheme (PPEAS) [27]. Routine internal quality control included the satisfactory analysis of manufacturer’s controls with each analytical run and the occasional inclusion of CDC control material when available.

In order to provide uniform confirmatory testing, an already functioning molecular laboratory at the National Institutes (NIH), University of the Philippines, Manila was chosen to provide follow-up testing using CE and molecular methods. Following reporting of NBS findings to the newborn’s primary care physician, the parent(s) are informed of the results and, if appropriate, the newborn is referred to a hematology specialist for further medical intervention. Where needed, a liquid blood specimen can be submitted to the confirmatory laboratory. Such follow-up testing is considered to be a routine part of the screening algorithm and is performed at no cost to the newborn’s family.

## 4. Discussion

Hemoglobinopathy NBS has been a successful strategy for early identification and treatment of sickle cell disease, including Hb S/β-thalassemia, since the early days of newborn screening in the U.S. [24]. With increasing immigration, early detection of other hemoglobin disorders through NBS, including Hb H disease and Hb E/β-thalassemia, have become increasingly important in places like California, where the Asian population is growing [31,32,33]. Few NBS programs outside of the U.S. and Europe have included hemoglobinopathies as part of their screening panel [34,35], although there is increasing interest in its inclusion in African countries where the incidence of SCD is extremely high [36]. Hemoglobinopathy NBS also provides a unique and efficient way of detecting α-thalassemias at or near birth by detecting Hb Bart’s (γ_4_). Hb Bart’s is formed when there is an absence of sufficient α-chains in the fetus (α-thalassemia), but disappears shortly after birth as Hb H (β_4_) begins to form. The detection of Hb H disease syndromes is particularly important since life threatening anemia can develop in young infants with the more severe form of Hb H/Constant Spring. Early identification of Hb H/Constant Spring, and other non-deletional forms of Hb H disease, through the NBS allows for close follow-up and prompt delivery of care to reduce adverse outcomes in these cases. Additionally, early knowledge of the presence of Hb H can be helpful clinically when other less severe symptoms are present [17].

Expansion to include hemoglobinopathy NBS in the Philippines took approximately 2 years from the time discussions began until its successful implementation. Collaborations with developed NBS programs already including hemoglobinopathy NBS were essential. Hemoglobinopathy screening data on Filipino newborns in California provided necessary and sufficient information to convince policy makers of its value in an expanded Philippine NBS panel. The documented clinical importance of early detection of Hb H disease and other hemoglobinopathies known in Asian populations further led to informed discussions with the hematology community, with a particular emphasis on Hb E disease, α-thalassemias, β-thalassemias, and Hb Constant Spring [17,37,38,39,40,41].

Clinically, the α-thalassemias range in severity from asymptomatic to death (in the case of hydrops fetalis) and a wide-range of clinical severity also exists with the β-thalassemias [12,17,37,38,39,40,41]. In the case of Hb H disease, the most prevalent α-thalassemia in Filipinos, NBS allows for the detection of Hb Bart’s (indicative of Hb H disease), which is only present in the early newborn period before gamma hemoglobin disappears as human hemoglobin production matures. In this case, NBS laboratory efficiency requires the establishment of an appropriate cut-off value for the quantification of Hb Bart’s so that unnecessary follow-up of asymptomatic carriers is minimized. While NBS provides the opportunity to detect carriers for this and many other screened conditions, it is usually the case that carriers are reported without additional follow-up in order to conserve resources that are then focused on detecting and ameliorating clinically significant diseases.

**Figure 2:**
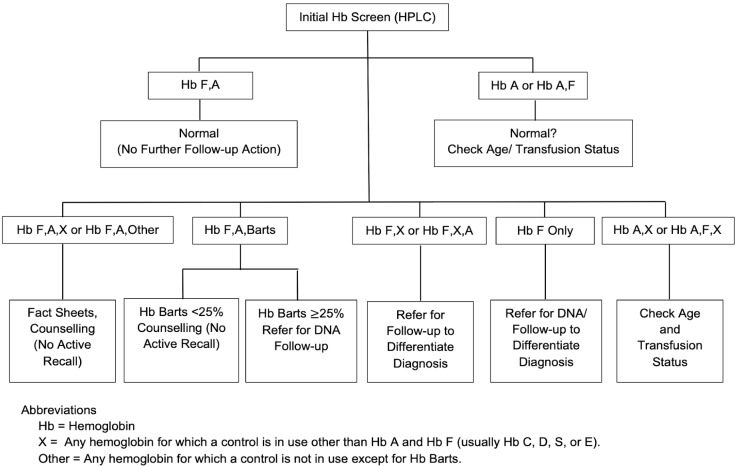
Flow diagram showing generalized analytical steps and interpretations in Philippine Hemoglobinopathy Newborn Screening.

As Hb degradation occurs with time after blood is absorbed and dried on filter paper, and concentrations of Hb Bart’s decrease rapidly after birth, percentages are dependent on the time of specimen collection and analysis. We chose to adopt the CNSP cut-off of 25% for Hb Bart’s based on their extensive studies in the past and the similarity of our methodology [13]. In their studies, Hb Bart’s level at birth was 35% (range, 25–49%) for Hb H disease with deletion of three α-globin genes and 41% (range, 35–52%) in cases of Hb H/Constant Spring disease. Thus, any patient with Hb Bart’s of 25% or higher is presumed to be at risk for Hb H disease and is referred for clinical follow-up (see Figure 2). The confirmatory laboratory is available for follow-up testing, including both CE and DNA analysis. Currently, case data are being accumulated for possible future publication and they will be periodically reviewed to see if cut-off adjustments are needed, as with all of our screened conditions.

**Figure 3:**
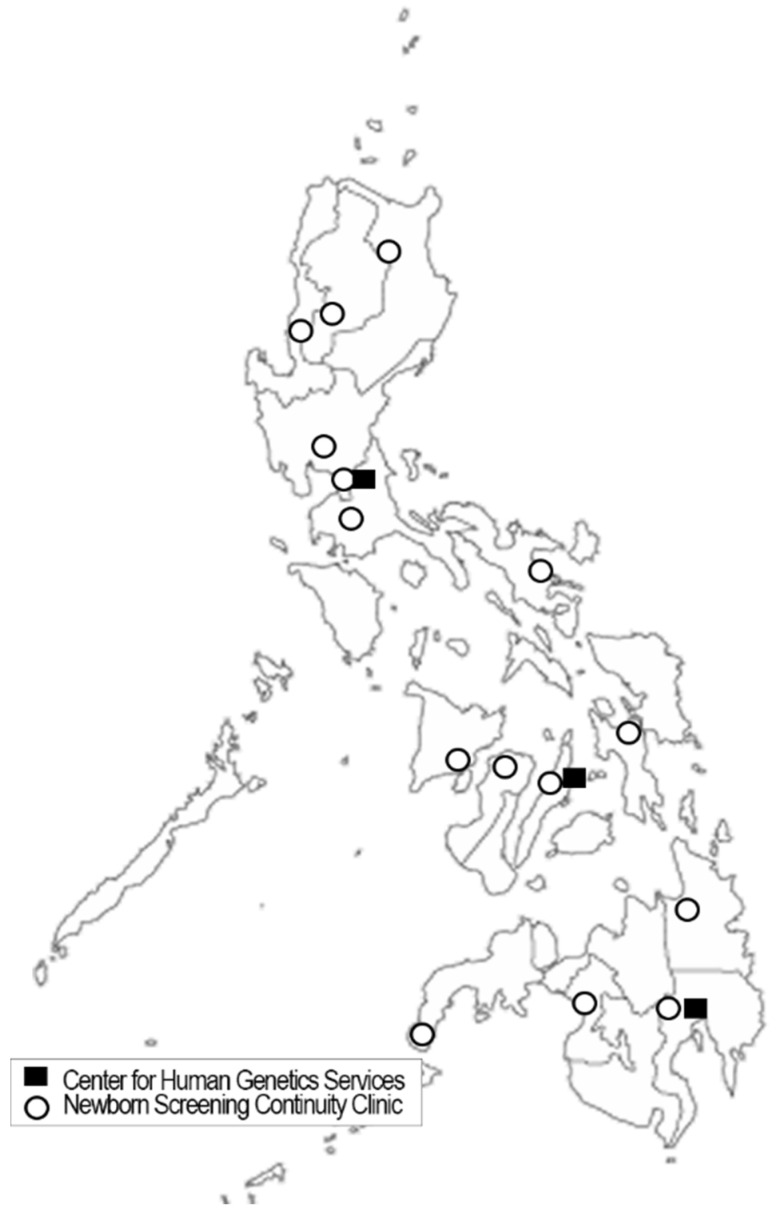
Distribution of Philippine Newborn Screening Continuity Clinics and Centers for Human Genetic Services. Centers for Human Genetic Services facilitate comprehensive clinical evaluation, appropriate management (diagnostic and therapeutic), and genetic counseling services to families or individuals with genetic conditions. Newborn Screening Continuity Clinics facilitate continuity of care of confirmed patients in its area of coverage.

The follow-up of all abnormal findings is aided by a network of 14 NBS continuity clinics (NBSCC), which include a medical follow-up team and are geographically dispersed across the country (see Figure 3). In the case of unusual or abnormal screening results, disorder fact sheets are provided to primary care physicians that provide basic information about the various hemoglobinopathies, including for those identified as probable disease carriers. While a genetic counselor is planned for each center, there are currently an insufficient number of counselors nationwide for complete staffing. A recently established Master of Science in Genetic Counselling program at the University of the Philippines Manila is expected to assist in providing trained genetic counselors in the future [42]. NBSCC clinical oversight and consultation is provided by three Centers for Human Genetic Services (CHGS). Each of the NBSCCs is directed by a medical geneticist knowledgeable in linking primary care physicians to regional disease specialists, covering diseases across the NBS spectrum. The CHGS not only provide an additional layer of medical specialization, but also assist in data accumulation and NBSCC oversight.

Following program implementation, consultative telephone conferences with outside experts were used to answer technical questions and to improve overall system operations. Details were added to the flow diagram (Figure 2) so that it could be more useful in the NSCs. To aid in harmonizing processes nationally, a dictionary of possible screening results was created, screening data being accumulated by NSCs, NBSCCs, and CHGSs were standardized, and laboratory and follow-up procedure manuals were harmonized. The screening equipment manufacturer provided training workshops and is providing long-term technical consultation. Screening laboratory technicians became adept at interpreting screening results, including reviewing screening patterns in order to validate the computerized result output, and all NSCs were enrolled in the CDC’s NSQAP. A protocol was established to resolve any discrepancies in results between the screening and confirmatory laboratories. After the third year of implementation, an expert review of the data was conducted in consultation with the Newborn Screening and Global Resource Center, USA, California Hemoglobinopathy Reference Laboratory, USA, and the Philippine Pediatric Hematology Society, Philippines. The results of this review, as with other consultations, allowed for the identification of issues and solutions that continue the ongoing process of program quality improvement. Included in the review were items such as clarification of the primary intent of screening to detect and address clinically significant disorders versus carriers, clarification of use of the abbreviation A2 as the name of a peak in the commercial HPLC tracing rather than the name of a specific hemoglobin (Hb A_2_), and recognition of the possibility for faster follow-up using filter paper specimens rather than whole blood (particularly important during the time of restricted patient travel as a result of COVID-19).

## 5. Conclusions

Hemoglobinopathies, including thalassemias, represent a significant group of disorders that can be detected, diagnosed, and treated early through NBS. The addition of hemoglobinopathy screening to the Philippine NBS panel was a multi-step process that began with the collection of population incidence data and evaluation of the possible costs and benefits. The overall purpose of NBS is to identify newborns at increased risk for disorders that are asymptomatic at birth and for which early diagnosis and treatment can positively impact the health of the child, the well-being of the family, and society in general. In the Philippine population, both α- and β-thalassemias are present and their clinical outcomes are being significantly and positively affected by inclusion of hemoglobinopathies in the expanded NBS panel.

While parents were initially charged a small fee in order to take advantage of the expanded NBS panel, payment for this panel was included in the national insurance package in 2019 [43]. As a result, expanded screening uptake continues to increase nationally, exceeding 90% of all newborns in 7400+ hospitals and birthing centers nationwide, prior to the COVID-19 pandemic. The goal is to implement full population NBS for all conditions now on the screening panel as quickly as possible. To this end, there is a robust quality improvement system in place that assesses coverage data and develops strategies for identifying and overcoming obstacles to quality NBS, including both screening and follow-up. Additionally, it includes case specific data collection at both the regional and national levels that assist in assessing the impact of all screening tests, including the incidence and outcome of the various hemoglobin disorders being detected [27]. Thus, while implementing or expanding NBS in a country with a developing economy is challenging, we have demonstrated that it can be successfully accomplished with thoughtful planning, careful implementation, and continuing collaboration with other programs.

## Figures and Tables

**Table 1:** Hemoglobin disorders in Filipino newborns—California vs. Philippines (adapted from Reference [11]).

Hemoglobin Disorders	^a^ Cases in Filipino Newborns Born in California 7 July 2005–6 July 2011	^a^ Prevalence in Filipino Newborns Born in California 7 July 2005–6 July 2011	^b^ Estimated Annual Cases of Filipino Newborns Born in Philippines
Hb H Disease	93	1:1195	1860
α Thalassemia Major	5	1:22,225	100
Homozygous E/E	3	1:37,042	60
Hb H/Constant Spring Disease	2	1:55,564	40
Hb SC Disease	2	1:55,564	40
Hb E/β^+^ Thalassemia	1	1:111,127	20
β Thalassemia major	1	1:111,127	20
Hb Variant/β^+^ Thalassemia	1	1:111,127	20
Hb SS Disease	1	1:111,127	20
Totals	109	1:1020	2240

^a^ Detected as part of California newborn screening program (*n* = 111,127). Parents included: Filipino-Filipino (61,088); Filipino-White (18,546); Filipino-Hispanic (8507); Filipino-Hispanic-White (3849); Filipino-Other (19,127); ^b^ assuming 100% coverage of 2 million annual births; overall prevalence (109 cases in 111,127 births).

## Abbreviations

α	Alpha
β	Beta
CAH	Congenital adrenal hyperplasia
CDC	Centers for Disease Control and Prevention (U.S.)
CE	Capillary electrophoresis
CH	Congenital hypothyroidism
CHGS	Center for Human Genetic Services
CNSP	California Newborn Screening Program (U.S.)
DOH	Department of Health (national health agency - Philippines)
G6PD	Glucose-6-phosphate dehydrogenase
GAL	Galactosemia
Hb	Hemoglobin
HCY	Homocystinuria
HPLC	High performance liquid chromatography
MSUD	Maple syrup urine disease
NBS	Newborn bloodspot screening
NBSCC	Newborn screening continuity clinic (long-term follow-up - Philippines)
NNSGRC	National Newborn Screening and Global Resource Center (U.S.)
NSC	Newborn Screening Center (screening laboratory - Philippines)
NSF	Newborn Screening Facility (screening specimen collection site - Philippines)
NSQAP	Newborn Screening Quality Assurance Program (U.S.)
NSRC	Newborn Screening Reference Center (Philippines)
PhilHealth	Philippine Health Insurance Corporation (national health insurance)
PKU	Phenylketonuria
POGS	Philippine Obstetrical and Gynecological Society
PPS	Philippine Pediatric Society
PT	Proficiency testing
QC	Quality control
SCD	Sickle cell disease

## References

[1] Padilla C., Domingo C.F. (2002). Implementation of newborn screening in the Philippines. Phil. J. Pediatr..

[2] David-Padilla C., Basilio J.A., Oliveros Y.E. (2009). Newborn Screening: Research to Policy. Acta Med. Philipp..

[3] Padilla C. (2003). Newborn screening in the Philippines. Southeast Asia J. Trop. Med. Pub. Health.

[4] Department of Health Memorandum No. 2012-0154. Inclusion of MSUD in the Newborn Screening Panel of Disorders. https://www.newbornscreening.ph/images/stories/ResourcesDOHPolicies/doh-2012-0154.pdf.

[5] Presidential Proclamation No. 540. Declaring the 1st Week of October of Each Year as “National Newborn Screening Week”. https://www.newbornscreening.ph/images/stories/ResourcesDOHPolicies/Presidential%20Proclamation%20No.%20540.pdf.

[6] Republic Act 9288—Newborn Screening Act of 2004. https://www.doh.gov.ph/sites/default/files/policies_and_laws/RA09288.pdf.

[7] PhilHealth Circular, No. 34, s-2006. PhilHealth Newborn Care Package. https://www.philhealth.gov.ph/circulars/2006/circ34_2006.pdf.

[8] American Academy of Pediatrics, Newborn Screening Task Force (2000). Serving the family from birth to the medical home—Newborn screening: A blueprint for the future. Pediatrics.

[9] American College of Medical Genetics, Newborn Screening Expert Group (2006). Newborn screening: Toward a uniform screening panel and system. Genet. Med..

[10] Feuchtbaum L., Carter J., Dowray S., Currier R.J., Lorey F. (2012). Birth prevalence of disorders detectable through newborn screening by race/ethnicity. Genet. Med..

[11] Padilla C.D. (2013). Enhancing Case Detection of Selected Inherited Disorders through Expanded Newborn Screening in the Philippines. Acta Med. Philipp..

[12] Therrell B.L., Hoppe C., Mann M.Y., Azimi M., Brants A., Brown S.E., Carte L.S., Dorle M.C., Eckman J.R., Flamini M. (2019). Newborn Screening for Hemoglobinopathies.

[13] Lorey F., Cunningham G., Vichinsky E.P., Lubin B.H., Witkowska H.E., Matsunaga A., Azimi M., Sherwin J., Eastman J., Farina F. (2001). Universal Newborn Screening for Hb H Disease in California. Genet. Test..

[14] Michlitsch J., Azimi M., Hoppe C., Walters M.C., Lubin B., Lorey F., Vichinsky E. (2009). Newborn screening for hemoglobinopathies in California. Pediatr. Blood Cancer.

[15] Hoppe C.C. (2011). Newborn Screening for Hemoglobin Disorders. Hemoglobin.

[16] Therrell B., Pass K., Therrell B. (1993). Hemoglobinopathy screening laboratory techniques for newborns. Laboratory Methods for Neonatal Screening.

[17] Lal A., Goldrich M.L., Haines D.A., Azimi M., Singer S.T., Vichinsky E.P. (2011). Heterogeneity of Hemoglobin H Disease in Childhood. N. Engl. J. Med..

[18] Piel F.B., Weatherall D.J. (2014). The α-thalassemias. N. Engl. J. Med..

[19] Hall J.G., Yuen J., Hunt J.A., Chu B.M., Hsia Y.E. (1991). Gene frequencies for the alpha-globin variants among Chinese, Laotians and Filipinos. Am. J. Hum. Genet..

[20] Ko T.M., Hwa H.L., Liu C.W., Li S.F., Chu J.Y., Cheung Y.P. (1999). Prevalence study and molecular characterization of alpha-thalassemia in Filipinos. Ann. Hematol..

[21] Ko T.M., Caviles A.P., Hwa H.L., Liu C.W., Hsu P.M., Chung Y.P. (1998). Prevalence and molecular characterization of beta-thalassemia in Filipinos. Ann. Hematol..

[22] Loeber J.G., Burgard P., Cornel M.C., Rigter T., Weinreich S.S., Rupp K., Hoffmann G.F., Vittozzi L. (2012). Newborn screening programmes in Europe; arguments and efforts regarding harmonization. Part 1—From blood spot to screening result. J. Inherit. Metab. Dis..

[23] Burgard P., Rupp K., Lindner M., Haege G., Rigter T., Weinreich S.S., Loeber J.G., Taruscio D., Vittozzi L., Cornel M.C. (2012). Newborn screening programmes in Europe; arguments and efforts regarding harmonization. Part 2—From screening laboratory results to treatment, follow-up and quality assurance. J. Inherit. Metab. Dis..

[24] Benson J.M., Therrell B.L. (2010). History and current status of newborn screening for hemoglobinopathies. Semin Perinatol..

[25] Hoppe C.C. (2009). Newborn screening for non-sickling hemoglobinopathies. Hematology.

[26] Shafer F.E., Lorey F., Cunningham G.C., Klumpp C., Vichinsky E., Lubin B. (1996). Newborn screening for sickle cell disease: 4 years of experience from California’s newborn screening program. J. Pediatr Hematol Oncol..

[27] Padilla C.P., Therrell B.L., Panol K.A.R., Concordia R.N., Reyes M.E.L., Jomento C.M., Maceda E.B.G., Lising J.A.C., Beltran F.D.E., Orbillo L.L. (2020). Philippine Performance Evaluation and Assessment Scheme (PPEAS): Experiences in newborn screening system quality control. Int. J. Neonatal Screen..

[28] Lorey F., Cunningham G., Shafer F., Lubin B., Vichinsky E. (1994). Universal Screening for Hemoglobinopathies Using High-Performance Liquid Chromatography: Clinical Results of 2.2 Million Screens. Eur. J. Hum. Genet..

[29] Liao C., Zhou J.-Y., Xie X.-M., Tang H.-S., Li R., Li D.-Z. (2013). Newborn Screening for Hb H Disease by Determination of Hb Bart’s Using the Sebia Capillary Electrophoresis System in Southern China. Hemoglobin.

[30] De Jesus V.R., Mei J.V., Bell C.J., Hannon W.H. (2010). Improving and assuring newborn screening laboratory quality worldwide: 30-year experience at the Centers for Disease Control and Prevention. Semin. Perinatol..

[31] Lorey F. (2000). Asian Immigration and Public Health in California: Thalassemia in Newborns in California. J. Pediatr. Hematol..

[32] Vichinsky E.P., Macklin E.A., Waye J.S., Lorey F., Olivieri N.F. (2005). Changes in the Epidemiology of Thalassemia in North America: A New Minority Disease. Pediatrics.

[33] Benz E.J. (2011). Newborn screening for α-thalassemia–keeping up with globalization. N. Engl. J. Med..

[34] Bain B.J. (2009). Neonatal/newborn haemoglobinopathy screening in Europe and Africa. J. Clin. Pathol..

[35] Therrell B.L., Padilla C.D., Loeber J.G., Kneisser I., Saadallah A., Borrajo G.J., Adams J. (2015). Current status of newborn screening worldwide: 2015. Semin. Perinatol..

[36] Therrell B.L., Lloyd-Puryear M.A., Ohene-Frempong K., Ware R.E., Padilla C.D., Ambrose E.E., Barkat A., Ghazal H., Kiyaga C., Mvalo T. (2020). Empowering newborn screening programs in African countries through establishment of an international collaborative effort. J. Community Genet..

[37] Chui D., Fucharoen S., Chan V. (2003). Hemoglobin H disease: Not necessarily a benign disorder. Blood.

[38] Fucharoen S., Viprakasit V. (2009). Hb H disease: Clinical course and disease modifiers. Hematology.

[39] Fucharoen S., Winichagoon P. (2011). Hemoglobinopathies in southeast Asia. Indian J. Med. Res..

[40] Vichinsky E. (2012). Advances in the treatment of alpha-thalassemia. Blood Rev..

[41] Fucharoen S., Weatherall D.J. (2012). The Hemoglobin E Thalassemias. Cold Spring Harb. Perspect. Med..

[42] Laurino M.Y., Padilla C.D. (2013). Genetic Counseling Training in the Philippines. J. Genet. Couns..

[43] PhilHealth Circular, No. 2018-0021. Enhancement of PhilHealth Newborn Care Package. https://www.philhealth.gov.ph/circulars/2018/circ2018-0021.pdf.

